# Deep Learning in Coeliac Disease: A Systematic Review on Novel Diagnostic Approaches to Disease Diagnosis

**DOI:** 10.3390/jcm12237386

**Published:** 2023-11-29

**Authors:** Kassem Sharif, Paula David, Mahmud Omar, Yousra Sharif, Yonatan Shneor Patt, Eyal Klang, Adi Lahat

**Affiliations:** 1 Department of Gastroenterology, Sheba Medical Centre, Ramat Gan 52621, Israel; zokadi@gmail.com; 2 Department of Internal Medicine B, Sheba Medical Centre, Ramat Gan 52621, Israel; paula.rdavid@gmail.com (P.D.); yopatt123@gmail.com (Y.S.P.); 3 Faculty of Medicine, Tel Aviv University, Tel Aviv 69978, Israel; mahmudomar70@gmail.com (M.O.);; 4 Department of Internal Medicine C, Haddasah Medical Centre, Hebrew University of Jerusalem, Jerusalem 9112102, Israel; yousras@hadassah.org.il; 5 Division of Data-Driven and Digital Medicine (D3M), Icahn School of Medicine at Mount Sinai, New York, NY 10029, USA; 6 The Charles Bronfman Institute of Personalized Medicine, Icahn School of Medicine at Mount Sinai, New York, NY 10029, USA; 7 ARC Innovation Center, Sheba Medical Center, Tel Hashomer, Ramat Gan 52621, Israel

**Keywords:** coeliac disease, deep learning, machine learning, innovation, diagnosis

## Abstract

Background: Coeliac disease affects approximately 1% of the global population with the diagnosis often relying on invasive and time-demanding methods. Deep learning, a powerful tool in medical science, shows potential for non-invasive, accurate coeliac disease diagnosis, though challenges remain. Objective: This systematic review aimed to evaluate the current state of deep-learning applications in coeliac disease diagnosis and identify potential areas for future research that could enhance diagnostic accuracy, sensitivity, and specificity. Methods: A systematic review was conducted using the following databases: PubMed, Embase, Web of Science, and Scopus. PRISMA guidelines were applied. Two independent reviewers identified research articles using deep learning for coeliac disease diagnosis and severity assessment. Only original research articles with performance metrics data were included. The quality of the diagnostic accuracy studies was assessed using the QUADAS-2 tool, categorizing studies based on risk of bias and concerns about applicability. Due to heterogeneity, a narrative synthesis was conducted to describe the applications and efficacy of the deep-learning techniques (DLT) in coeliac disease diagnosis. Results: The initial search across four databases yielded 417 studies with 195 being removed due to duplicity. Finally, eight studies were found to be suitable for inclusion after rigorous evaluation. They were all published between 2017 and 2023 and focused on using DLT for coeliac disease diagnosis or assessing disease severity. Different deep-learning architectures were applied. Accuracy levels ranged from 84% to 95.94% with the GoogLeNet model achieving 100% sensitivity and specificity for video capsule endoscopy images. Conclusions: DLT hold substantial potential in coeliac disease diagnosis. They offer improved accuracy and the prospect of mitigating clinician bias. However, key challenges persist, notably the requirement for more extensive and diverse datasets, especially to detect milder forms of coeliac disease. These methods are in their nascent stages, underscoring the need of integrating multiple data sources to achieve comprehensive coeliac disease diagnosis.

## 1. Introduction

Coeliac disease, an immune-mediated disorder activated by the consumption of gluten in genetically predisposed individuals, constitutes a concerning global health issue, affecting approximately 1% of the global population [1]. Despite substantial progress in understanding the pathogenesis of this condition, several areas remain unexplored. These include the diagnostic challenges in heterogeneous presentations, treatment for patients unresponsive to gluten sensitivity, and the utility of serology tests as standalone diagnostics without the need for pathology-based confirmation [2]. Currently, the diagnosis of coeliac disease hinges on serological tests and histological confirmation derived from invasive duodenal endoscopic biopsies [3,4,5]. However, these procedures are invasive, labor-intensive, and time-demanding. Several biopsies are required to achieve sufficient sensitivity for a definitive diagnosis [1,6].

The integration of deep-learning techniques (DLT) in the realm of medical science, particularly within the context of coeliac disease diagnosis and management, has gained significant attention. Deep learning, a subfield of machine learning, has displayed remarkable potential in analyzing complex medical data, including endoscopic images, to discern significant patterns and diagnostic markers [7,8].

The expansive reach of artificial intelligence (AI) has significantly transformed various domains of medical science, extending well beyond the diagnosis of coeliac disease. In cancer diagnostics, AI-enhanced algorithms have been employed to improve prostate cancer detection with some approaches achieving diagnosis accuracies as high as 98% by fusing particle swarm optimization with neural networks [9,10]. In the fight against infectious diseases like malaria, innovative diagnostic models utilizing advanced capsule networks have proven to be more rapid and precise compared to conventional microscopic evaluation [11]. Within medical informatics, sophisticated methods such as artificial neural networks now assist healthcare professionals in making intricate diagnostic decisions, indicating a transition from traditional rule-based systems to sophisticated, data-driven models. In the field of hematology, deep-learning methods have excelled in the accurate identification of B cell acute lymphoblastic leukemia, leading to the creation of mobile applications that facilitate a more streamlined diagnostic workflow [12]. These advancements highlight the pivotal role of AI in enhancing various medical diagnostics, heralding a new era of more effective, accurate, and patient-focused diagnostic procedures.

Deep-learning algorithms have been developed for the examination of video capsule endoscopy images, offering a non-invasive and notably accurate way of diagnosing coeliac disease [13,14]. Moreover, deep-learning techniques, such as convolutional neural networks and Fisher encoding, have exhibited promise in the classification of endoscopic images and the detection of villous atrophy, a hallmark of coeliac disease [15,16].

The integration of AI into medical diagnostics has shown promising results with deep-learning models revealing significant potential in the early detection and classification of diseases. The initial outcomes present a compelling case for AI’s role in enhancing diagnostic accuracy, leading to optimized patient outcomes. However, the journey from experimental application to routine clinical practice is complex and multifaceted. Therefore, it is imperative to establish the scope and efficacy of these technologies systematically. This systemic review, therefore, has been undertaken to comprehensively examine the current findings in the literature to delineate the practicality and implications of deep learning within this specific medical context.

## 2. Methods

### 2.1. Search Strategy

This systematic review study was prospectively registered at PROSPERO (with registration number: CRD42023474948 on 23 October 2023) and was carried out according to the Preferred Reporting Items for Systematic Reviews and Meta-Analyses (PRISMA) guidelines.

A comprehensive and systematic search was conducted across four prominent databases, PubMed, Embase, Web of Science, and Scopus, up until October, 2023. The objective was to identify original research articles that applied DLT for the diagnosis or assessment of coeliac disease severity using endoscopy images. For the search, a combination of keywords related to “coeliac disease”, “deep learning”, “machine learning”, “neural networks”, “endoscopy”, and “videocapsule” were used. The specific search strings for each database are detailed in the Appendix A.

### 2.2. Study Selection

#### 2.2.1. Inclusion and Exclusion Criteria

In order to maintain the relevance and specificity of the chosen research studies, we included in our systematic review exclusively original research articles that focused on the application of deep-learning methods for either coeliac disease diagnosis or the evaluation of its severity using endoscopic images and that provided data for the assessment of deep-learning model performance metrics, including accuracy, sensitivity, and specificity. All reviews, case reports, conference abstracts, and studies conducted in languages other than English were excluded.

#### 2.2.2. Data Extraction

A meticulous data extraction process was adopted. Two independent reviewers were tasked with extracting pertinent information using a standardized form, ensuring consistency. The data points targeted for extraction included the first author’s name, year of publication, study design, sample size, the DLT employed, dataset details for model training and validation, performance metrics of the model, and the main findings. In instances where discrepancies arose between the reviewers, discussions were initiated to achieve consensus, and if needed, a third reviewer was consulted.

#### 2.2.3. Risk of Bias

To ascertain the quality and robustness of the methodologies adopted in the selected diagnostic accuracy studies, the QUADAS-2 (Quality Assessment of Diagnostic Accuracy Studies-2) tool was employed. This tool offers a systematic approach to evaluating the potential risk of bias and concerns regarding applicability in diagnostic studies. It assesses four key domains: patient selection, index test, reference standard, and flow and timing. Based on the evaluations, studies were systematically categorized in terms of their risk of bias as high, low, or unclear. Concerns about the applicability of studies were similarly categorized.

### 2.3. Data Analysis

Given the inherent heterogeneity among the DLT and the datasets utilized across studies, a meta-analysis was deemed unsuitable. Consequently, the data were synthesized in a narrative format, accentuating the applications and the efficacy of the different deep-learning models in diagnosing and assessing coeliac disease.

### 2.4. DLT Overview

This review focused on certain DLT that have showcased promise in the realm of medical imaging. These techniques include convolutional neural networks (CNNs), which are specifically tailored to process grid-like images, and residual learning, which uses skip connections to enhance the efficiency of deep neural networks. Additionally, Fisher encoding was also explored, a method adept at aggregating local descriptors into a singular vector, facilitating classification. A comprehensive exposition of these techniques and their potential applications in coeliac disease diagnosis is delineated in the subsequent sections of the review.

## 3. Results

### 3.1. Search Results and Study Selection

The initial search across the four databases yielded a total of 417 studies. These were distributed as follows: PubMed (72 studies), Embase (156 studies of which 92 were duplicates from PubMed, leaving 64 unique studies), Web of Science (71 studies), and Scopus (118 studies). After removing duplicates (195 studies), 222 studies remained for further scrutiny.

**Figure 1:**
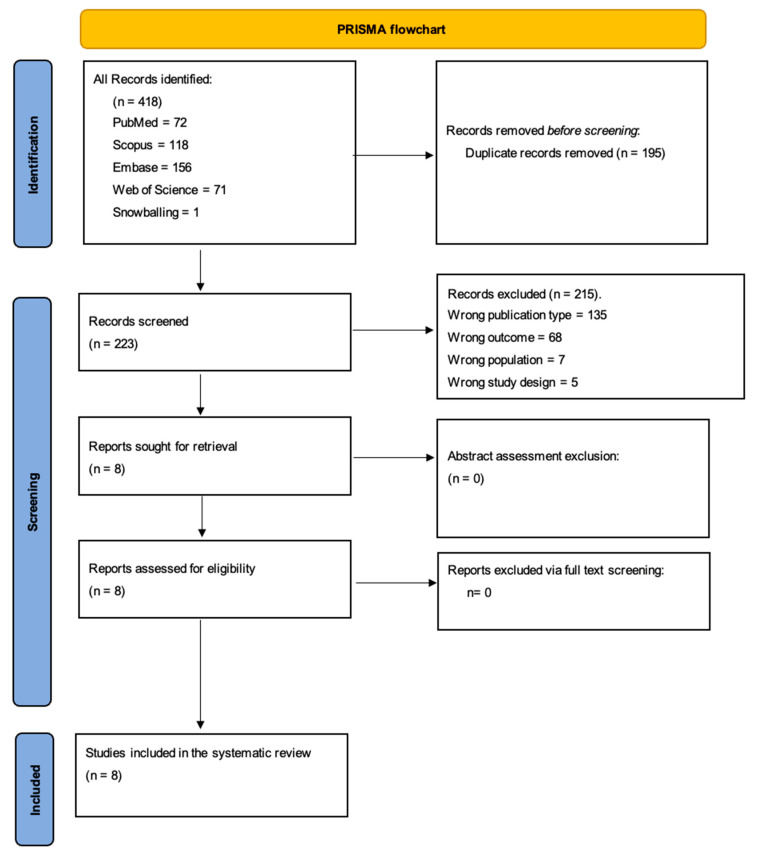
PRISMA flowchart.

Upon rigorous evaluation against the inclusion and exclusion criteria, a significant number of studies were excluded for the following reasons: wrong publication type (135 studies), wrong outcome (68 studies), wrong population (7 studies), and incorrect study design (5 studies). Additionally, one study was included through the snowballing method. Ultimately, eight studies were deemed suitable for inclusion in this systematic review (Figure 1 shows the PRISMA flowchart, and Table 1 summarizes the included studies).

### 3.2. Risk of Bias

**Figure 2:**
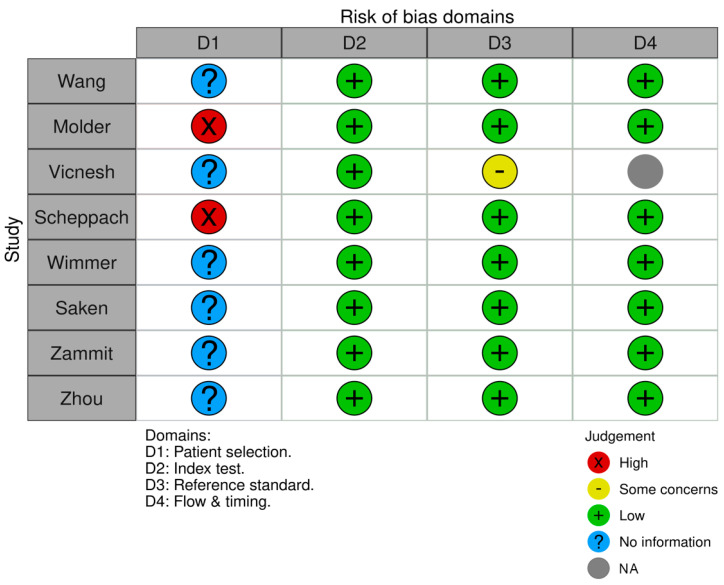
Distribution of risk of bias concerns across individual domains. Abbreviations—NA—not applicable.

**Figure 3:**
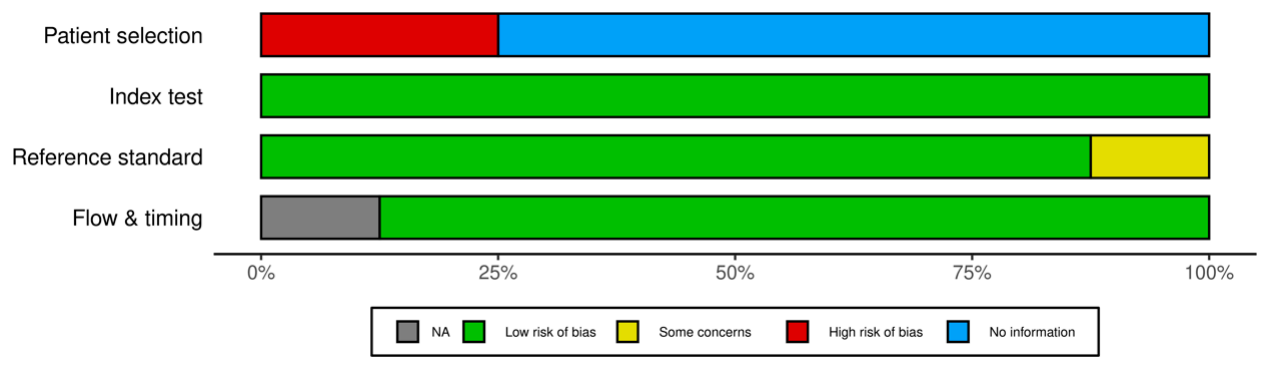
Cumulative assessment of overall risk of bias concerns across all included studies.

Utilizing the QUADAS-2 tool, we assessed the risk of bias across multiple domains: patient selection, the index test, reference standard, and flow and timing. For patient selection, the primary source of uncertainty stemmed from unclear methods of patient or image enrollment in several studies, notably in those by Wang, Vicnesh et al., Saken et al., and Zhou et al. [13,14,17,20]. However, most studies avoided a case-control design with notable exceptions in Molder et al. and Scheppach et al. [15,19]. Concerning the index test and reference standard, most studies showcased a low risk of bias with well-defined methodologies and consistent reference standards, particularly evident in studies by Wang et al., Molder et al., Scheppach et al., Wimmer et al., and Zhou et al. [13,14,15,16,19]. In terms of flow and timing, given the dataset-centric nature of many studies, traditional patient flow considerations were often not directly applicable, resulting in a general low risk of bias. Additionally, we assessed concerns about the applicability of the studies, categorizing them similarly as high, low, or unclear. Overall, while many studies demonstrated a commendable low risk of bias and applicability concerns, certain studies exhibited uncertainties, emphasizing the need for clearer patient selection methodologies in future research (Figure 2 and Figure 3).

### 3.3. Overview of Included Studies

The eight studies included in this review were published between 2017 and 2023. They predominantly focused on the utilization of DLT to diagnose coeliac disease or assess its severity using endoscopic imaging. Various deep-learning architectures were employed across the studies, including DAISY descriptors, GoogLeNet, ResNet50, Inception-v3, and ResNet18. Five of the studies specifically aimed at diagnosing coeliac disease using different methodologies, two centered on assessing disease severity, and one performed a comparative analysis between human experts and machine-learning algorithms in evaluating coeliac disease severity. The application of these techniques underscores the evolving landscape of coeliac disease research, emphasizing the increasing reliance on advanced computational methods for diagnosis and assessment.

### 3.4. Narrative Synthesis of Deep-Learning Approaches in Coeliac Disease Research

Advancements in machine learning (ML) and deep learning (DL) have catalyzed breakthroughs in the domain of coeliac disease research. This synthesis endeavors to elucidate the multifaceted DL techniques employed across various studies, providing a cohesive understanding of their applications and outcomes.

1. Harnessing video capsule endoscopy (VCE) images for diagnosis:

DAISY descriptors: Vicnesh et al. (2019) [17] introduced a computer-aided detection system employing DAISY descriptors to project 2D VCE images onto 1D vectors. Coupled with Shannon entropy for feature extraction and particle swarm optimization (PSO) for feature selection, this approach garnered an accuracy of 89.82% with a sensitivity of 94.35% and specificity of 83.20%.

GoogLeNet: Zhou et al. (2017) [14] embarked on a quantitative assessment of coeliac disease pathology in the small intestine by leveraging GoogLeNet, a deep convolutional neural network (DCNN), on preprocessed VCE clips. Astonishingly, the model realized 100% sensitivity and specificity. Furthermore, the introduced “evaluation confidence” measure shed light on the severity of mucosal lesions in the small bowel.

ResNet50 and Inception-v3: An innovative recalibration module was assimilated into these deep-learning algorithms by Wang et al. (2020) [13] to refine the diagnosis from VCE images. By capturing salient features in local channel feature maps, their recalibrated model attained an accuracy of 95.94% with a laudable sensitivity of 97.20%.

2. DLTTargeting duodenal endoscopy images:

Endoscopic imagery, offering a meticulous glimpse into the duodenal realm, has been pivotal for coeliac disease research when analyzed through DL.

Layered convolutional neural networks (CNN): Molder et al. (2023) [19] deployed ML and DL algorithms on duodenal endoscopy images, setting their sights on detecting villous atrophy (VA). Their layered CNN model emerged preeminent, boasting a sensitivity of 99.67% and a PPV of 98.07%.

ResNet18: Venturing into VA detection on endoscopic imagery, Scheppach et al. (2023) [15] employed ResNet18, a DL model, achieving a sensitivity of 90%, specificity of 76%, and an overall accuracy of 84%. Notably, the AI algorithm surpassed both endoscopy fellows and seasoned experts.

3. Hybrid techniques and comparative analyses:

The fusion of techniques and juxtaposition of methods have been paramount in refining coeliac disease diagnostic techniques.

Hybrid ML approaches: Saken et al. (2021) [20] crafted a synergetic methodology, integrating multilevel thresholding, discrete wavelet transform (DWT), and scale-invariant texture recognition for endoscopy images. This hybrid technique realized an accuracy of 94.79%, a sensitivity of 94.29%, and a specificity of 95.08%.

Fisher encoding of CNN activations: Wimmer et al. (2018) [16] steered their research toward the encoding of CNN activations, employing Fisher encoding for diagnosing coeliac disease and coeliac peptide (CP). Their method, when applied on three distinct CNN architectures coupled with support vector machines (SVMs), outshone other techniques and negated the need for target dataset training.

4. Comparative analysis for enhanced understanding:

Chetcuti Zammit et al. (2023) [18] orchestrated a comparative study that juxtaposed the prowess of human experts against a machine-learning algorithm (MLA) in assessing the severity of coeliac disease using VCE. The VCE imagery from patients underwent a meticulous evaluation by both human experts and the MLA. A commendable inter-reader agreement on coeliac villous damage was recorded at alpha = 0.924, underscoring the harmony between the machine-learning algorithm and the human evaluators.

### 3.5. Summary of Diagnostic Accuracies

Among the studies that utilized VCE images for coeliac disease diagnosis, Zhou et al. (2017) [14], using the GoogLeNet model, stood out with a perfect accuracy score of 100%. On the other hand, the method by Vicnesh et al. (2019) [17], which incorporated DAISY descriptors, reported an accuracy of 89.82%, marking the lower end of the spectrum. When focusing on duodenal endoscopy images, the study by Molder et al. (2023) [19], using layered convolutional neural networks (CNN), achieved a high sensitivity of 99.67%. Meanwhile, Scheppach et al. (2023) [15], using the ResNet18 model, reached a commendable overall accuracy of 84%. In the realm of hybrid techniques, the approach by Saken et al. (2021) [20], which combined multiple methodologies, achieved an accuracy nearing the top tier at 94.79%.

## 4. Discussion

The clinical presentation of coeliac disease exhibits significant heterogeneity and can sometimes present with unspecific symptoms, leading to frequent misdiagnosis or non-diagnosis [21,22]. Consequently, the utilization of artificial intelligence (AI) and machine/deep-learning algorithms for the identification of villous atrophy in duodenal endoscopic and video capsule imagery demonstrates significant potential, especially in situations where the initial procedures were conducted without suspicion of coeliac disease as the underlying condition, which could potentially lead to underdiagnosis [15].

Deep-learning methodologies have the capability to autonomously identify visual disparities in image frames between patients with coeliac disease and control subjects, consequently aiding in coeliac disease diagnosis. By enabling early detection of the condition, AI has the potential to prevent the progression to severe stages, possibly avoiding long-term complications and improving patient outcomes. Moreover, computer-aided approaches reduce clinician bias.

In examining the recent advancements in the application of deep learning for diagnosing coeliac disease, a notable variety in data type and methodology emerges. A spectrum of endoscopy images, ranging from video capsules to duodenal scans, has been employed in studies and subjected to sophisticated algorithms; notably, convolutional neural networks (CNNs) [19], distinguished for their adeptness in image classification, exhibit promise in discerning subtle mucosal changes indicative of coeliac disease. Techniques, such as the incorporation of DAISY descriptors and Shannon entropy, have enhanced the feature extraction process, a critical facet for nuanced differentiation between mucosal patterns associated with the disease and those observed in normal conditions [17].

Validation strategies in these investigations have predominantly concentrated on cross-validation techniques, thereby ensuring the resilience of the models against overfitting. While this methodological stringency substantiates the reliability of the models, it also accentuates a pivotal constraint: the imperative for diverse and expansive datasets. The prevalent reliance on cross-validation underscores the importance of a comprehensive dataset, thereby highlighting the exigency for inclusivity in terms of data diversity and volume.

Indeed, when machine and DLT were applied for the detection and diagnosis of coeliac disease based on duodenal endoscopic or video capsule imagery, accuracy levels ranged from 84% to 95.94% [13,15,16,17,19,20]. When compared to endoscopy fellows and experts, these AI approaches demonstrated superiority [15,18]. Notably, Zhou et al. achieved both 100% sensitivity and specificity in the assessment of coeliac disease pathology using GoogLeNet for video capsule imagery [14]; however, the power of this study was really low. This is particularly significant in the context of video capsule endoscopy, where concordance among blinded observers, especially those with limited experience, can be challenging [23,24].

Despite these encouraging strides, the utilization of deep learning in coeliac disease diagnosis remains in its early stages, necessitating the resolution of several challenges. These challenges encompass the requirement of larger and more diverse datasets for training and validation purposes along with the need for additional investigation into the potential role of deep learning in improving disease management [14,17]. The sample sizes in the currently available studies, though adequate, suggest a potential bias toward more pronounced manifestations of the disease.

For example, while the study by Zhou et al. [14] reports an impressive outcome with GoogLeNet, achieving 100% sensitivity and specificity in identifying coeliac disease from video capsule imagery, such metrics warrant a cautious interpretation due to the small sample size (*n* = 6 for confirmed coeliac disease cases). It is essential to consider the statistical power of the study, which is inherently limited in this context. The small number of cases may not provide a comprehensive representation of the diverse manifestations of coeliac disease, potentially leading to overfitting of the model to the dataset at hand. Furthermore, the absence of a larger validation cohort raises questions regarding the generalizability of these results. While the findings are encouraging, they highlight the need for larger-scale studies to validate the efficacy of GoogLeNet and similar AI tools in varied clinical scenarios. Ensuring a substantial sample size would improve the statistical power, thereby enhancing the confidence in the diagnostic accuracy of such advanced technologies when applied to a broader population. A similar comment could be made about the Molder et al. study with a similarly reduced power (*n* = 18 patients with CD). The high sensitivity may be directly related to the restricted power of the study [19].

The translation of the mentioned findings into real-world medical settings comes with challenges. When incorporating AI into the diagnostic process, it is important to consider how it affects day-to-day operations and finances, making sure it is seen as an adjunct to rather than a replacement for the nuanced clinical judgment of healthcare professionals. Moreover, the AI models must be subjected to continual validation against larger, multi-institutional datasets to iron out any biases and confirm their efficacy across diverse populations. Comparatively, the accuracy of deep-learning models is very different from the subjective interpretation used in histopathology, the current gold standard for coeliac disease diagnosis. While human assessments can vary, deep learning provides a consistency that might reduce discrepancies in diagnosis.

Regardless of technique, the potential contributions of AI to coeliac disease diagnosis, encompassing both enhanced accuracy and speed of diagnosis and potentially reduced workload, are undeniable. However, it is essential to recognize that we are still in the early stages of this endeavor. The development of larger databases for machine and deep learning is imperative for refining these algorithms. Furthermore, it should be emphasized that mild forms of coeliac disease, categorized as Marsh I (characterized by increased mucosal lymphocytes) and Marsh II (characterized by crypt proliferation) are often not detectable during macroscopic endoscopic and video capsule examinations, rendering them imperceptible to the majority of AI algorithms. Presently, ongoing research is dedicated to the automatic detection of Marsh classification types through the collection and analysis of a larger volume of video clips from patients with coeliac disease and control subjects [14]. However, to address this limitation and achieve better coverage of mild cases, new algorithms that integrate endoscopic/video capsule imagery with biopsy results as well as serologic and genetic data are required.

Our systematic review highlights that AI techniques represent significant progress in coeliac disease diagnosis, each with its unique strengths. However, it is not our intent to advocate for AI as the sole diagnostic tool, rather as an additional help. As mentioned, GoogLeNet, despite the study power limitation, stood out for its exceptional sensitivity and specificity in video capsule endoscopic-based diagnosis [14]. In duodenal endoscopy images, the layered CNN model achieved remarkable sensitivity [16], while ResNet18 displayed strong performance [14]. Hybrid approaches and Fisher encoding offered effective alternatives, emphasizing the diversity of methods in the field. The choice of technique may depend on the specific diagnostic requirements and available data. We acknowledge that, while AI applications show promise, they have limitations. The current AI models are dependent on the quality and diversity of the data they are trained on, and there is a risk of bias if the datasets are not representative of the broader patient population. Additionally, AI’s interpretability remains a challenge, as the decision-making process of complex algorithms is often opaque, which may impede clinical trust and acceptance. There are also concerns regarding the generalizability of these AI systems across different endoscopic equipment and imaging techniques. Further research is required to address these limitations, to refine the algorithms, and to assess the long-term clinical outcomes associated with the use of AI in the diagnosis of CD.

Our review maintains a balanced view, advocating for cautious optimism while emphasizing the need for ongoing evaluation and validation of AI technologies in diverse clinical settings.

### Future Directions

This systematic review underscores the potential of deep learning in the advancement of CD diagnostics. Future studies should focus on expanding and diversifying datasets to encompass a wider spectrum of the disease’s clinical presentations, including subtle and atypical manifestations. The integration of deep learning with other diagnostic modalities, like serology and genetics, may provide a more holistic diagnostic framework. Additionally, cross-disciplinary collaborations could lead to the development of more robust, generalizable models that consider variability across different endoscopic equipment and imaging techniques. The pursuit of algorithm interpretability will also be crucial to foster clinical trust and facilitate the translation of these technologies into routine clinical practice. Moreover, with the promising preliminary results, a pathway toward the automation of the Marsh classification of coeliac disease is conceivable. As AI methodologies continue to evolve, their potential to streamline diagnostic processes and reduce clinician workload could be significant, necessitating ongoing evaluation to ensure they complement rather than replace clinical expertise. Lastly, the ethical implications and accessibility of AI in healthcare settings, particularly in resource-limited environments, warrant thoughtful consideration to ensure equitable advancements in patient care.

## 5. Conclusions

Deep-learning techniques, such as convolutional neural networks and hybrid approaches, show significant promise in coeliac disease diagnosis. They offer enhanced accuracy and have the potential to reduce clinician bias. However, challenges include the need for larger and more diverse datasets, especially to detect mild coeliac disease forms. These methods are still in their early stages, and the integration of multiple data sources is essential for comprehensive coeliac disease diagnosis.

## Figures and Tables

**Table 1:** Studies included in the review.

No.	Author, Year, Ref	Main Objective	Data Type	Methodology	Validation Type	Sample Size	Main Results
1	Vicnesh et al., 2019 [17]	Develop CAD for coeliac from capsule images	Endoscopy Images	DAISY descriptors, Shannon entropy, PSO	10-fold cross-validation	Total number of video clips: 52 with coeliac disease (CD), 55 healthy. Total number of images used for analysis: 2140 (1100 healthy mucosa, 1040 damaged mucosa).	89.82% accuracy, 89.17% PPV, 94.35% sensitivity, 83.20% specificity.
2	ChetcutiZammit et al., 2023 [18]	Compare CD severity assessment with VCE by expert human readers and an MLA.	VCE Images	Scoring by humans and MLA, 4-point scale	Primary validation with 36 VCE test set, ensembled prediction across orientations, and curve fit post-processing.	Total patients: 34 (18 with coeliac disease, 16 controls) Total images: 66 from coeliac disease patients, 16 from controls	Inter-reader agreement on coeliac villous damage, alpha = 0.924. Excellent MLA agreement with expert readers.
3	Wang et al., 2020 [13]	Develop a deep-learning module to diagnose coeliac disease from VCE images.	VCE Images	Recalibration in ResNet50, Inception-v3	10-fold cross-validation	Total participants: 37 (21 with coeliac disease, 16 healthy individuals)	95.94% accuracy, 97.20% sensitivity, 95.63% specificity.
4	Molder et al., 2023 [19]	Automated detection of endoscopic markers during routine endoscopy examinations for COELIAC DISEASE diagnosis.	Endoscopy Images	ML, DL on images, histology reference	train–validation split	Total patients: 505 (182 with villous atrophy, 323 controls) Total images: 1704 (858 from patients with villous atrophy, 846 from controls)	Layered CNN best performance: 99.67% sensitivity and 98.07% PPV.
5	Scheppach et al., 2023 [15]	Apply AI for the macroscopic detection of VA during EGD to improve diagnostic performance.	Endoscopic Images	Trained ResNet18 on 858 images	5-fold cross-validation	Total patients: 87 (11 with coeliac disease, 76 controls) Total images: 330	AI algorithm: 90% sensitivity, 76% specificity, and 84% accuracy. Outperformed endoscopy fellows and experts.
6	Saken et al., 2021 [20]	Enhance the diagnostic accuracy of COELIAC DISEASE using CAD systems in endoscopy.	Endoscopy Images	Hybrid ML, multilevel thresholding, DWT	10-fold cross-validation	Total patients: 353 Total images: 1661 (986 healthy mucosa, 675 affected by coeliac disease)	94.79% accuracy, 94.29% sensitivity, 95.08% specificity.
7	Wimmer et al., 2018 [16]	Automate the diagnosis of CD and CP using Fisher encoding of [14,16] activations.	Endoscopy Images	Fisher on CNN activations, SVMs	5-fold cross-validation	Total patients: 63 with biopsy-confirmed coeliac disease	Approach outperformed other CNN- and non-CNN-based approaches and required no training on the target dataset.
8	Zhou et al., 2017 [14]	Establish a DCNN for quantitative assessment of pathology in the small intestine from videocapsule endoscopy.	VCE Clips	GoogLeNet on preprocessed clips	7-fold cross-validation	Total participants: 21 (11 with coeliac disease, 10 controls)	100% sensitivity and specificity for the testing set. Evaluation confidence may relate to the severity level of small bowel mucosal lesions.

CAD: Computer-Aided Detection, CD: Coeliac Disease, VCE: Video Capsule Endoscopy, MLA: Machine Learning Algorithm, DAISY: Digital Automated Identification System, PSO: Particle Swarm Optimization, PPV: Positive Predictive Value, ML: Machine Learning, DL: Deep Learning, CNN: Convolutional Neural Network, EGD: Esophagogastroduodenoscopy, VA: Villous Atrophy, DWT: Discrete Wavelet Transform, SVMs: Support Vector Machines, DCNN: Deep Convolutional Neural Network, ResNet50: Residual Network 50, Inception-v3: Inception Version 3, GoogLeNet: Google’s Deep Neural Network, CP: Coeliac Peptide.

## Data Availability

Studies are published online.

## Appendix A. The Systematic Literature Review Boolean Strings

Pubmed—(“celiac disease” OR “coeliac disease”) AND (“deep learning” OR “machine learning” OR “neural networks” OR “convolutional neural networks” OR “CNN” OR “artificial intelligence”).

Embase—(‘celiac disease’ OR ‘coeliac disease’) AND (‘deep learning’ OR ‘machine learning’ OR ‘neural networks’ OR ‘convolutional neural networks’ OR ‘CNN’ OR ‘artificial intelligence’) AND (‘diagnosis’ OR ‘diagnostic’ OR ‘endoscopy’ OR ‘endoscopic images’ OR ‘videocapsule’ OR ‘villous atrophy’).

Web of science—(“celiac disease” OR “coeliac disease”) AND (“deep learning” OR “machine learning” OR “neural networks” OR “convolutional neural networks” OR “CNN” OR “artificial intelligence”) AND (“diagnosis” OR “diagnostic” OR “endoscopy” OR “endoscopic images” OR “videocapsule” OR “villous atrophy”).

Scopus—(TITLE-ABS-KEY(“celiac disease” OR “coeliac disease”) AND TITLE-ABS-KEY(“deep learning” OR “machine learning” OR “neural networks” OR “convolutional neural networks” OR “CNN” OR “artificial intelligence”) AND TITLE-ABS-KEY(“diagnosis” OR “diagnostic” OR “endoscopy” OR “endoscopic images” OR “videocapsule” OR “villous atrophy”)).
